# β-blocker eye drops affect ocular surface through β2 adrenoceptor of corneal limbal stem cells

**DOI:** 10.1186/s12886-021-02186-w

**Published:** 2021-12-05

**Authors:** Xingyue Yuan, Xiubin Ma, Lingling Yang, Qingjun Zhou, Ya Li

**Affiliations:** 1 Medical College, Qingdao University, Qingdao, China; 2 State Key Laboratory Cultivation Base, Shandong Provincial Key Laboratory of Ophthalmology, Eye Institute of Shandong First Medical University, 5 Yan’erdao Road, Qingdao, 266071 China

**Keywords:** β-Blocker eye drops, β2-adrenoceptor, Corneal, Limbal stem cells, Wound healing

## Abstract

**Background:**

Topical application of β-blocker eye drops induces damage to the ocular surface in clinical. However, the mechanism involved remains incompletely understood. The purpose of this study was to investigate the influence and mechanism of β-blocker eye drops on corneal epithelial wound healing.

**Methods:**

Corneal epithelial wound healing models were constructed by epithelial scraping including in the limbal region and unceasingly received eye drops containing 5 mg/mL β-blocker levobunolol, β1-adrenoceptor (β1AR)-specific antagonist atenolol or β2-adrenoceptor (β2AR)-specific antagonist ICI 118, 551. For the migration assay, the murine corneal epithelial stem/progenitor cells (TKE2) were wounded and subsequently incubated with levobunolol, atenolol, or ICI 118, 551. The proliferation and colony formation abilities of TKE2 cells treated with levobunolol, atenolol, or ICI 118, 551 were investigated by CCK-8 kit and crystal violet staining. The differentiation marker Cytokeratin 3 (CK3), the stem cell markers-Cytokeratin 14 (CK14) and Cytokeratin 19 (CK19), and corneal epithelium regeneration-related signaling including in Ki67 and the phosphorylated epithelial growth factor receptor (pEGFR) and phosphorylated extracellular signal-regulated kinase 1/2 (pERK1/2) were assessed by immunofluorescence staining.

**Results:**

Levobunolol and ICI 118, 551 impaired corneal wound healing, decreased the expressions of CK3, CK14, and CK19 after limbal region scraping in vivo and reduced the migration and proliferation of TKE2 in vitro, whereas atenolol had no significant effect. Moreover, levobunolol and ICI 118, 551 inhibited corneal wound healing by mediating the expression of Ki67, and the phosphorylation of EGFR and ERK1/2 in the limbal and regenerated corneal epithelium.

**Conclusion:**

β-blocker eye drops impaired corneal wound healing by inhibiting the β2AR of limbal stem cells, which decreased corneal epithelial regeneration-related signaling. Therefore, a selective β1AR antagonist might be a good choice for glaucoma treatment to avoid ocular surface damage.

## Background

Toxic keratitis involves complex clinical manifestations, which is difficult to differentiate from other ocular surface diseases, and requires a careful medical history and clinical examination. The characteristic features of toxic keratitis mainly include superficial punctuate or epithelial defects, ulceration, scarring, pannus, neovascularization, or perforation [1]. Common drugs that cause toxic keratitis include preservatives in ophthalmic medications, such as benzalkonium chloride; topical anesthetic agents, such as proparacaine, benoxinate, and tetracaine; antiviral agents, such as trifluridine; antibiotics, such as vancomycin and gentamicin; cycloplegic agents, such as atropine; and glaucoma medications. Glaucoma is associated with progressive visual loss, usually caused by an increase in intraocular pressure (IOP), making glucoma the second leading cause of blindness worldwide. In clinical situations, the β-blockers levobunolol and timolol, the carbonic anhydrase inhibitor dorzolamide, parasympathomimetic pilocarpine, and sympathomimetics such as dipivefrin and apraclonidine are frequently used for glaucoma treatment [2]. The treatment of glaucoma is long and is usually combined with other drugs. Thus, these factors increase the risk of toxic keratitis. Therefore, research on drug toxicity may help prevent permanent damage to the ocular surface.

Adrenoceptors (ARs), which belong to the G protein-coupled receptor superfamily, mediate diverse effects at all sites throughout the body [3]. βARs, which are expressed by trabecular meshwork cells, ciliary bodies, microvessels, and optic nerve tissue, are involved in glaucoma pathogenesis by regulating the secretion and outflow of aqueous humor in the eyes [4–6]. βAR antagonists have multiple functions, including increasing airway resistance by blocking the bronchiolar smooth muscle, stimulating the lipolysis of fat cells and glycogenolysis in the liver, and inducing the release of renin in the kidney and angiotensin in the circulation [7]. Mechanically, the topical application of β-blocker eye drops blocks the sympathetic nerve endings in the ciliary epithelium, thereby decreasing aqueous humor production, which then reduces the IOP. Currently, the β-blocker eye drops levobunolol (0.5%) and timolol (0.5%) are first-choice agents that are used alone or with other medications to treat open-angle glaucoma or ocular hypertension by reducing the high pressure of the eyes [7–11]. However, clinical studies have identified that the topical application of β-blocker eye drops has side effects on goblet cell density [12, 13], corneal tear film [14–16], and the human ocular surface [17–20]. The topical application of β-blocker eye drops decreases the corneal epithelial barrier function [18] and delays corneal epithelial wound healing [19, 20]. Myungsik et al. found that the use of β-blocker eye drops showed a significant reduction in the corneal epithelial thickness [21]. Moreover, the corneal epithelial cells are derived from limbal epithelial stem cells which support the renewal of the corneal epithelium [22]. Rodolfo et al. investigated the morphologic changes of the corneoscleral limbus in glaucoma patients using laser scanning confocal microscopy and impression cytology. They found that β-blockers presented a worse limbal transition epithelium regularity, which suggested the antiglaucoma therapy might potentially disturb the environment of limbal stem cells [23]. Therefore, the damage to the limbal stem cells exhibits more serious consequences than the damage to the differentiated corneal cells, with reference to wound healing/regeneration. However, further investigation is needed to determine which type of epithelial cells and primary ARs are influenced by β-blocker eye drops.

In the present study, we investigated the effects of the anti-glaucoma agent levobunolol on corneal limbal stem cells by establishing corneal wound healing models with the entire corneal epithelium, including limbal region scrape. Atenolol is a selective β1AR blocker, and ICI 118,551 is a highly selective β2AR antagonist, which is used widely for hypertension, angina pectoris, and for the regulation of pulmonary systolic pressure [24, 25].

Furthermore, the primary AR inhibited by levobunolol has been identified using β1AR-specific antagonist atenolol and β2AR-specific antagonist ICI 118, 551 in the corneal wound healing model. Kawakita et al. separated a mouse corneal epithelial progenitor cell line (TKE2) from CD-1 albino mice and successfully constructed a tissue engineered corneal epithelium [26]. Qu et al. identified that TKE2, which expresses the ATP-binding cassette subfamily G member 2, Ki67, proliferating cell nuclear antigen, P63, shares similar characteristics to the murine limbal stem cells and can be used to study the mechanism of the limbal epithelium [27]. Therefore, TKE2 was used to explore the primary ARs influenced by β-blocker eye drops in vitro. We also investigated the differentiation marker Cytokeratin 3 (CK3), the stem cell markers Cytokeratin 14 (CK14) and Cytokeratin 19 (CK19), and corneal epithelial regeneration-related signaling to illustrate the mechanism by which β-blockers impaired corneal wound healing.

## Materials and methods

### Animal models

The experimental study included 141 C57BL/6 mice with an age of 8 weeks and a body weight of 20-25 g at baseline. The Medical Ethics Committee of Shandong Eye Institute approved the study, and the ARVO Statement and the ARRIVE Guidelines for the use of animals in ophthalmic and vision research were employed. The animals were purchased from Beijing Vital River Laboratory Animal Technologies Co. Ltd. in Beijing, China. All animals were sacrificed by cervical dislocation, and the eyes were extirpated immediately for further research.

### Corneal epithelial wound healing

The mice were systemically anesthetized with pentobarbital sodium (50 mg/kg, ip) and topically anesthetized with 2% xylocaine. Then, the corneal epithelium was marked with 2.5 mm or 3.0 mm trephine and scraped with Alger Brush II corneal rust ring remover (Alger Co., Lago Vista, TX). Ofloxacin was used to prevent postoperative infection. The experimental groups unceasingly received eye drops containing 5 mg/mL of levobunolol (MedChemExpress, Princeton, NJ), atenolol (MedChemExpress) or ICI 118, 551 (MedChemExpress) for 4 times a day and mice received saline as vehicle control. The defects of corneal epithelium were visualized every 12 h by staining with fluorescein sodium and photographed with BQ 900 slit lamp (Haag-Streit, Bern, Switzerland). The experiment was carried out including five mice for each group and repeated at least three times. The defect area was analyzed by Image J software [28].

### Cell culture

The TKE2 cells, which were kindly presented by Dr. Tetsuya Kawakita (Keio University, Tokyo, Japan), were cultured in keratinocyte serum-free medium (KSFM, GibcoThermo Fisher Scientific Inc., Waltham, MA) and supplemented with human keratinocyte growth supplement (HKGS, Gibco) and recombinant epidermal growth factor (EGF, 5 ng/mL, R&D systems Inc., Minneapolis, MN).

### Cell migration analysis

The TKE2 cells were trypsinized and plated into the 12-well plates until confluence. Then the TKE2 cells were starved with KSFM in the presence of EGF (5 ng/mL). After starvation for 24 h, the TKE2 cells were wounded with a micropipette tip and subsequently incubated with 50 μM levobunolol, atenolol, or ICI 118, 551 for another 24 h, and PBS was used as control. The photographs of wound closure were used for migration quantification.

### Cell proliferation analysis

The TKE2 cells were trypsinized and plated into the 96-well plates (2000 per well) and the TKE2 cells were incubated with 50 μM levobunolol, atenolol, or ICI 118, 551 for 48 h, and PBS was used as control. Then, the proliferation abilities of TKE2 cells treated by levobunolol, atenolol, or ICI 118, 551 were investigated by cell counting kit-8 (CCK-8, Bioss, Beijing, China) and the absorbance of each well was measured at 450 nm by using a microplate reader (Model 680; Bio-199 Rad, Hercules, CA).

### Colony formation

The TKE2 cells were trypsinized and plated into the 12-well plates (800 per well) and then the TKE2 cells were incubated with levobunolol (50 μM), atenolol (10 μM), or ICI 118, 551 (10 μM) for 7 days, and PBS was used as control. Then, the Colony formation abilities of TKE2 cells treated by levobunolol, atenolol, or ICI 118, 551 were investigated by crystal violet staining solution (Beyotime, Shanghai, China) and the images were observed using an Echo microscope (Echo Laboratories, San Diego, CA).

### Immunofluorescence staining

Mouse eyeballs were excised and snap-frozen in Tissue-Tek OCT compound at 2 or 3 days after levobunolol, atenolol, or ICI 118, 551 treatment. The frozen sections (7 μm) were fixed in 4% paraformaldehyde for 15 min, permeabilized with 0.1% Triton X-100 for 15 min, and then blocked with 5% BSA for 1 h at room temperature. The sections were then incubated with anti-CK3 (Abcam, Cambridge, MA), anti-CK14 (Abcam), anti-CK19 (Proteintech, Chicago, IL), anti-Ki67 (Abcam), anti- phosphorylated epithelial growth factor receptor (pEGFR, Abcam), or anti-phosphorylated extracellular signal-regulated kinase 1/2 (pERK1/2, Cell Signaling Technology, Danvers, MA) overnight at 4 °C and subsequently incubated with fluorescein-conjugated secondary antibody (Santa Cruz Biotechnology, Santa Cruz, CA) for 1 h, followed by 4′, 6-diamidino-2-Phenylindole (DAPI, Solarbio, Beijing, China) staining for 5 min. All stainings were observed through an Eclipse TE2000-U microscope (Nikon, Tokyo, Japan) and were quantified by Image J software. Firstly, the images were put on, then, which were conversed to 8 bit and adjusted to gray scale followed by measuring parameters including the area, mean gray value (Mean), integral optical density. Finally, we got average gray scale of these images which were used to statistically analyze.

### Statistical analysis

The statistical analysis was performed with GraphPad Prism 8.0 and SPSS 24.0. The statistical significance was analyzed by Student’s t-test and one-way ANOVA. A *P* value less than 0.05 was considered statistically significant, a *P* value less than 0.01 was considered extremely significant.

## Results

### Non-selective β-blocker levobunolol impairs corneal wound healing through limbal stem cells in mice

**Fig. 1:**
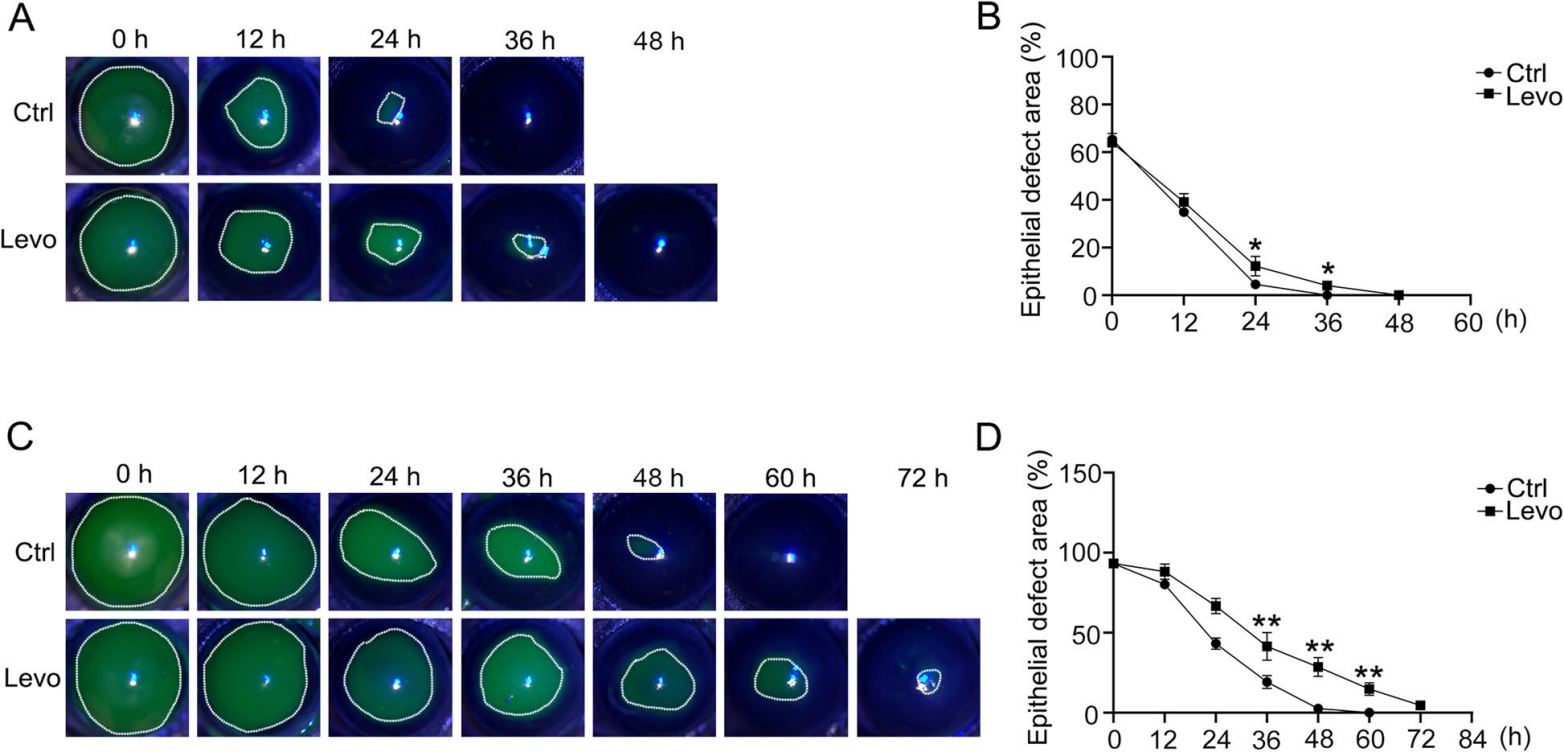
Effects of non-selective β-blocker levobunolol on corneal epithelial wound healing in mice. **A** The central region of corneal epithelium marked with 2.5 mm trephine was scraped and treated with saline (Ctrl) and non-selective β-blocker levobunolol (Levo), and then stained with fluorescein sodium every 12 h. **B** A graph of the epithelial defect area at 12 h was presented as the percentage of the original wound. **C** The central and limbal regions of the corneal epithelium with 3.0 mm trephine were scraped and treated with saline (Ctrl) and levobunolol (Levo), and then stained with fluorescein sodium at 12 h. **D** A graph of the epithelial defect area was presented as the percentage of the original wound. Data are representative of means ± SD. *P < 0.05, **P < 0.01

**Fig. 2:**
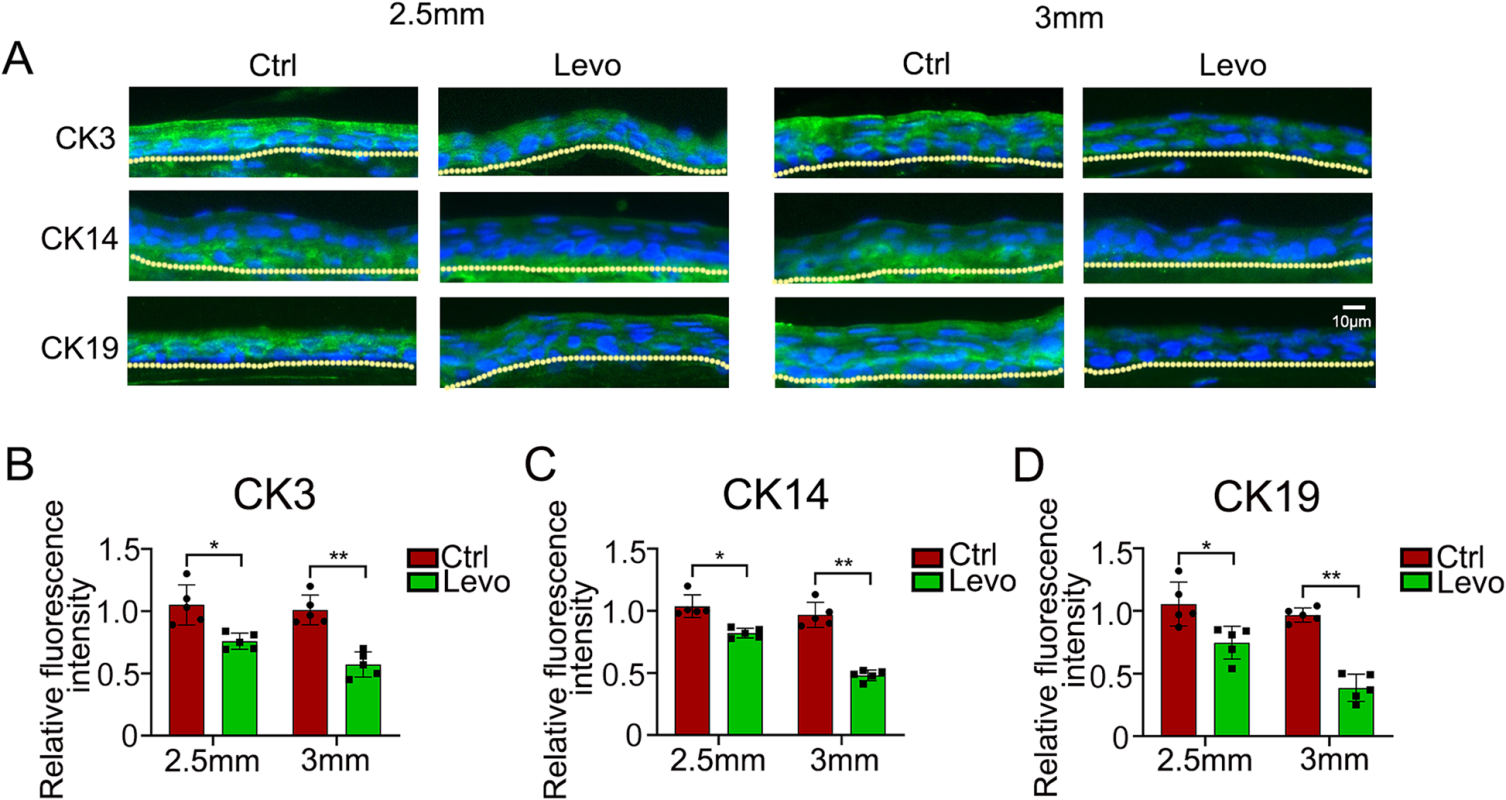
The expressions of CK3, CK14 and CK19 in the corneal limbal epithelium after levobunolol treatment. The corneal epithelium was scraped with 2.5 or 3 mm trephine and unceasingly received eye drops of saline (Ctrl) and levobunolol (Levo) for 4 times a day. At 2 or 3 days post wounding, the expressions of CK3 (**A**), CK14 (**A**), and CK19 (**A**) in the corneal limbal epithelium were detected by immunofluorescence staining. Moreover, the quantification of stained sections was carried out using the Image J software (**B-D**). Data are representative of means ± SD. *P < 0.05, **P < 0.01

To assess the influence of β-blocker eye drops on corneal wound healing, we removed the central or the entire corneal epithelium including the limbal region by marking with 2.5 or 3 mm trephine. Approximately 5 mg/mL β-blocker eye drops levobunolol, which was equivalent to the concentration used clinically, was then applied topically to the mice, and wound healing was observed using fluorescein sodium staining. After central region debridement, the corneal epithelial healing rate of the levobunolol-treated group exhibited a marginally significant difference compared with the control group (Fig. 1A, B). However, after the corneal limbal region was scraped, the difference between the levobunolol and control groups was highly significant throughout the wound healing process (Fig. 1C, D). To investigate the recovery in the 2.5- and 3-mm eye wounds, we detected the expressions of the differentiation marker CK3, and the stem cell markers CK14 and CK19 in the limbal region. The results showed that the levobunolol-treated group expressed weaker CK3, CK14, and CK19 intensities than that in the control group in both 2.5- and 3-mm eye wounds. Moreover, the difference in CK3, CK14, and CK19 expressions in 3-mm eye wound was more significant compared with that in the 2.5-mm eye wound (Fig. 2A-D). These results indicated that levobunolol was more influential in the corneal limbal stem cells than in the central epithelium.

### β2AR antagonist inhibits corneal wound healing through limbal stem cells in mice

**Fig. 3:**
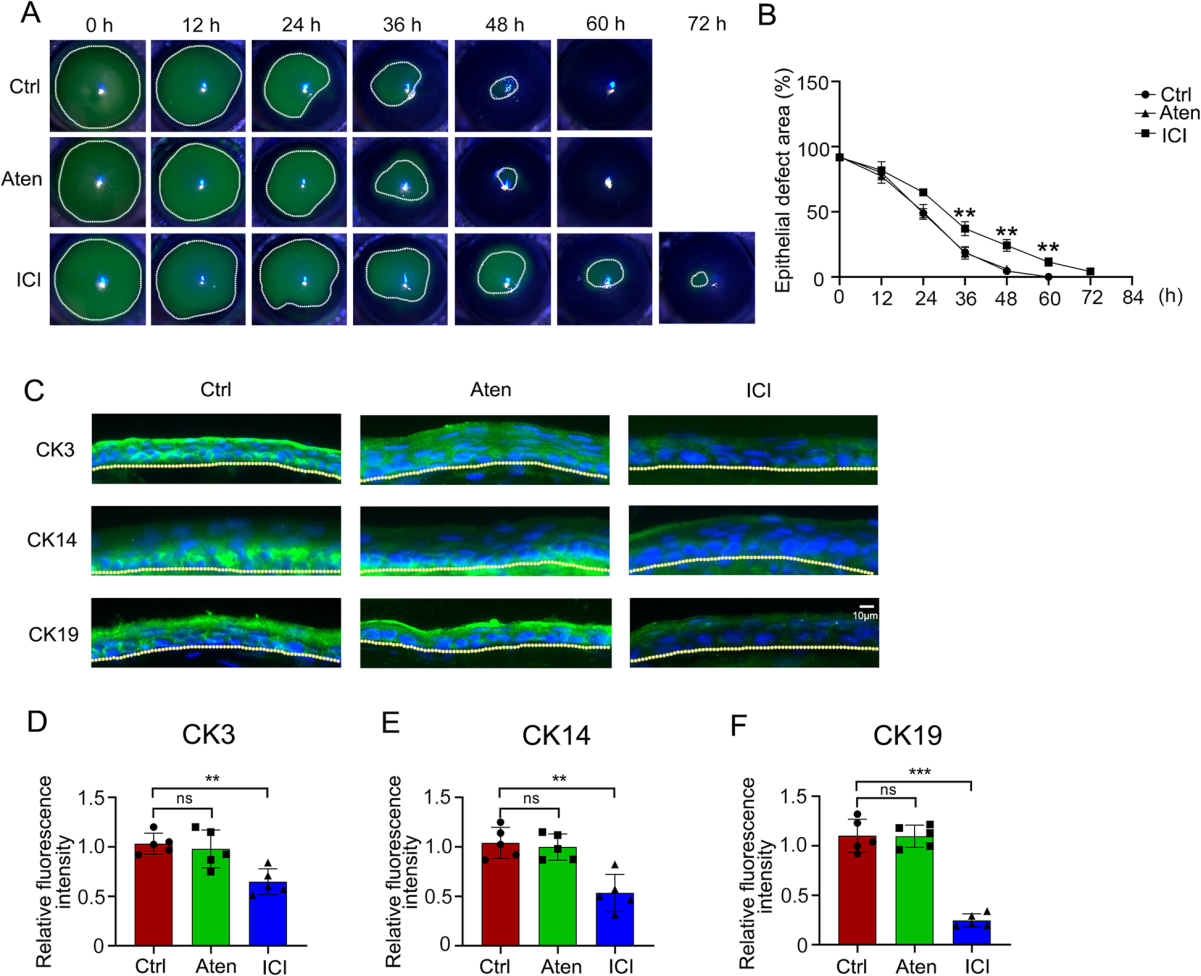
Effects of β1AR and β2AR antagonists on corneal epithelial wound healing and expressions of CK3, CK14, and CK19 in the corneal limbal epithelium. **A** The corneal epithelium was scraped with 3.0 mm trephine and treated with saline (Ctrl), the β1AR inhibitor atenolol (Aten), and the β2AR inhibitor ICI 118, 551 (ICI), and then stained with fluorescein sodium every 12 h. **B** A graph of the epithelial defect area was presented as the percentage of the original wound. The expressions of CK3 (**C**), CK14 (**C**), and CK19 (**C**) in the corneal limbal epithelium were detected by immunofluorescence staining. Moreover, the quantification of stained sections was carried out using the Image J software (**D-F**). Data are representative of means ± SD. **P < 0.01, ***P < 0.001

To delineate the primary AR antagonized by the non-selective β1 and β2AR inhibitor levobunolol during corneal wound healing, the β1AR specific antagonist atenolol or β2AR specific antagonist ICI 118, 551 was applied topically to the mice after the entire corneal epithelium, including the limbal region, was removed. The results showed that the wounded area of mice treated with ICI 118, 551 was significantly larger than that in the control and atenolol groups (Fig.3A, B). The CK3, CK14, and CK19 expressions in the atenolol- or ICI 118, 551-treated groups were then detected using immunofluorescence staining. The results showed that the fluorescence intensities of CK3, CK14, and CK19 were diminished in the ICI 118, 551-treated group, whereas no significant change was observed in the atenolol group compared with the control group (Fig. 3C-F). This finding illustrated that the β-blocker eye drops levobunolol delayed wound healing mainly due to the inhibition of β2AR of limbal stem cells.

### β2AR antagonist mediates the migration and proliferation of TKE2 cells

**Fig. 4:**
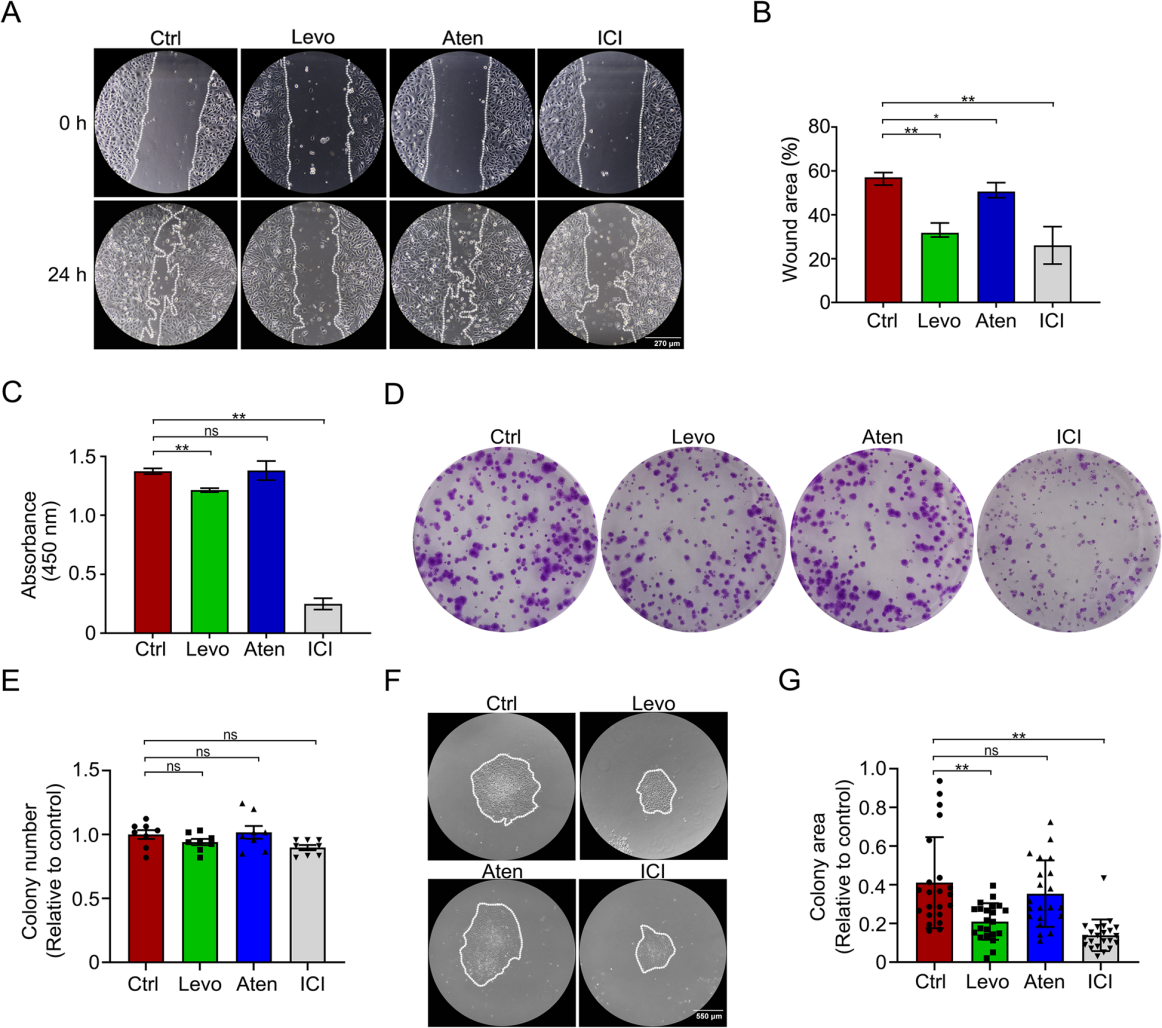
Effects of the non-selective β-blocker levobunolol, the β1AR antagonist atenolol, and the β2AR antagonist ICI 118, 551 on the migration and proliferation of TKE2 cells. **A** Migration of TKE2 cells replenished with PBS, levobunolol, atenolol, or ICI 118, 551 for 24 h was observed. **B** Migration of TKE2 cells was analyzed using the Image J software. **C** Proliferation of TKE2 cells treated with PBS, levobunolol, atenolol, or ICI 118, 551 for 48 h was detected by CCK-8. Colony formation (**D**) and colony number (**E**) of TKE2 cells treated with PBS, levobunolol, atenolol, or ICI 118, 551 for 7 days. **F** Colony area of TKE2 cells treated with PBS, levobunolol, atenolol, or ICI 118, 551 for 7 days. **G** The colony areas were analyzed using the Echo microscope software. Data are representative of means ± SD. *P < 0.05, **P < 0.01

It was revealed that levobunolol had more influence on corneal limbal stem cells, which resulted in the delay of corneal wound healing. To investigate the effects of β-blocker eye drops in regulating the corneal wound healing in vitro, TKE2 cells, which shared a similar characteristic to the murine limbal stem cells, were chosen to conduct further study [27]. Firstly, the monolayer of TKE2 cells was wounded and incubated with levobunolol, atenolol, or ICI 118, 551 for 24 h, and the results revealed that the migration capacity of levobunolol-and ICI 118, 551-treated TKE2 cells was significant reduced while atenolol-treated TKE2 cells exhibited a marginally reduction compared to the control (Fig. 4A, B). As expected, the proliferation rates of levobunolol-and ICI 118, 551-treated TKE2 cells were significantly lower than the control group while atenolol-treated TKE2 cells were equivalent to the control levels (Fig. 4C). These results illustrated that β-blocker eye drops impaired the migration and proliferation of the limbal stem cells by inhibiting the β2AR.

The colony forming of TKE2 treated with levobunolol, atenolol, or ICI 118, 551 for 7 days were compared by crystal violet staining and the results showed that there was no significant difference in the clone number of the control group and levobunolol, atenolol, or ICI 118, 551 groups (Fig. 4D, E). However, the size of the clones treated by levobunolol or ICI 118, 551 was significantly smaller than the control group (Fig. 4F, G). It indicated that β-blocker eye drops reduced the proliferation of limbal stem cells without impacting the clone formation rate.

### Inhibition of β2AR alters corneal epithelial regeneration-related signaling

**Fig. 5:**
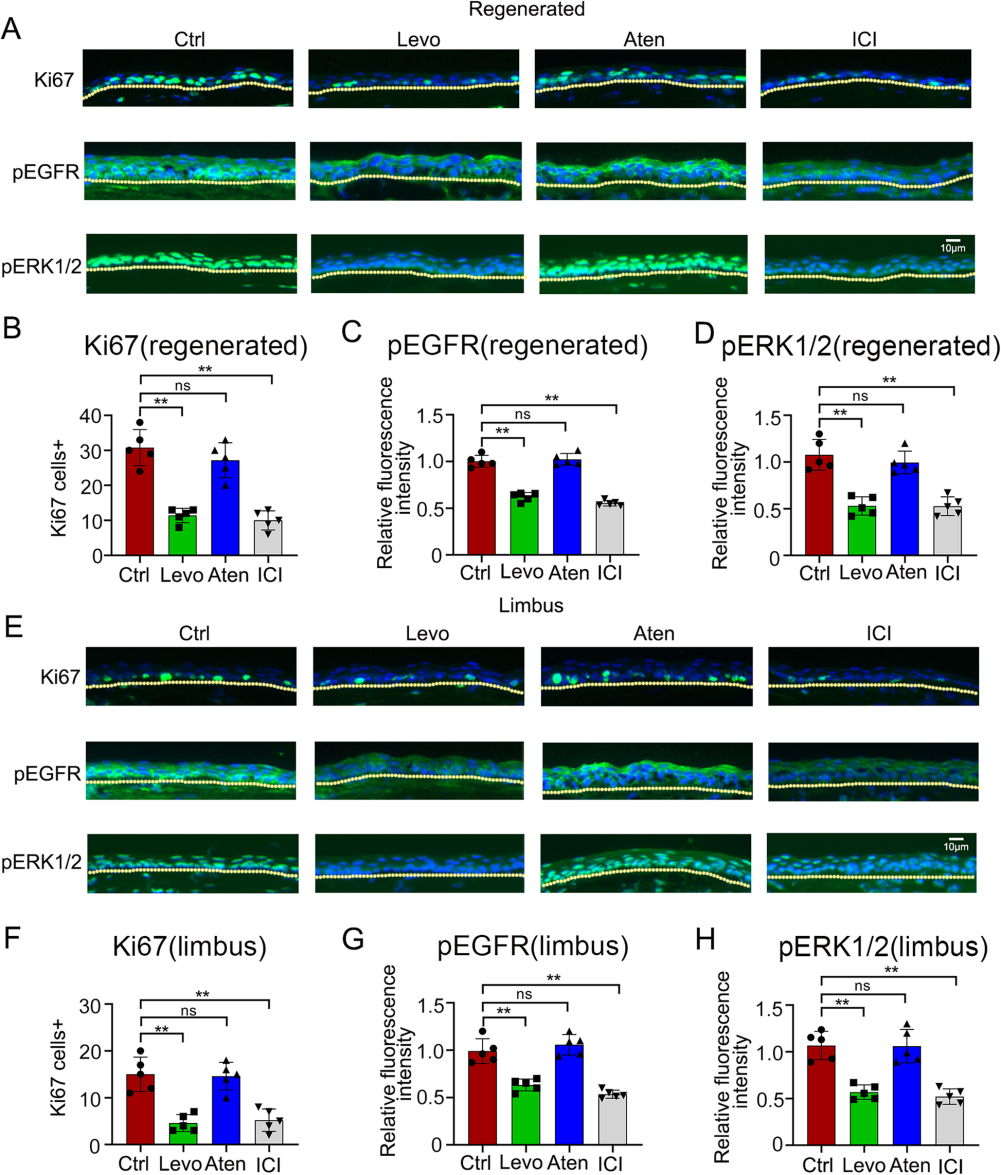
Effects of the non-selective β-blocker levobunolol, the β1AR antagonist atenolol, and the β2AR antagonist ICI 118, 551 on corneal epithelial regeneration-related signaling. The corneal epithelium was scraped including the limbal region and unceasingly received eye drops of saline (Ctrl), levobunolol (Levo), atenolol (Aten), or ICI 118, 551 (ICI) for 4 times a day. At 3 days post wounding, the expression of Ki67 (**A**, **E**), and the phosphorylation of EGFR (**A**, **E**) and ERK1/2 (**A**, **E**) in the corneal epithelium including in the regenerated and limbal regions, were detected by immunofluorescence staining. Moreover, the quantification of the stained sections was carried out using the Image J software (**B-D**, **F-H**). Data are representative of means ± SD. **P < 0.01

To elucidate the critical signaling pathways mediated by the β-blocker eye drops during corneal wound healing, we investigated the proliferation marker Ki67, and the phosphorylation of EGFR and ERK1/2 in the limbal and regenerated corneal epithelium. As shown in Fig. 5A, E, the Ki67 positive cells diminished after levobunolol and ICI 118, 551 treatments compared with that in the control mice. Moreover, the staining density of pEGFR and pERK1/2 was also weakened in the levobunolol- and ICI 118, 551-treated groups in both the regenerated and limbal regions (Fig. 5A, E). The fluorescence intensity in the corneal epithelium was quantified by the Image J software (Fig. 5B-D, F-H). The results suggested that the β-blocker eye drops altered the corneal epithelial regeneration-related signaling mainly due to the antagonized β2AR.

## Discussion

βAR, which is expressed by trabecular meshwork cells, ciliary bodies, microvessels, and optic nerve tissue, is involved in glaucoma pathogenesis [4–6]. Moreover, the expression of β2AR is dominant compared with that of β1AR in the cornea, however, minimal β3AR expression was found [29]. Using the corneal epithelial wound healing models, we demonstrated that the β-blocker eye drops levobunolol and the β2AR antagonist ICI 118, 551 impaired the wound healing of mice that were scraped, including in the limbal region, whereas the β1AR antagonist atenolol showed no significant effects. Meanwhile, levobunolol inhibited the migration and proliferation of TKE2 through β2AR, but not through β1AR. Furthermore, levobunolol and ICI 118, 551 impaired corneal epithelial wound healing, and exhibited decreased expressions of CK3, CK14, CK19, Ki67 and phosphorylated EGFR and ERK1/2. Thus, our results demonstrated that the β-blocker eye drop levobunolol impaired corneal wound healing through the inhibition of β2AR of the limbal stem cells, resulting in a decrease in corneal epithelial regeneration-related signaling.

βAR regulates multiple cellular processes, such as cell proliferation, cell motility, inflammation, angiogenesis, and apoptosis [30]. Recent evidence suggests that βAR agonist isoproterenol could promote cell proliferation through activation of β2AR, while pre-treatment with ICI 118, 551 blocks isoproterenol-induced cell growth in the A549 cells [30, 31]. Meanwhile, isoprenaline also accelerates the recovery of intestinal stem cells after cancer treatment with chemotherapeutic agents, which are mediated by β2AR [32]. In addition, noradrenaline and β2AR selective agonist salmeterol stimulate the proliferation of neural precursor cells, which have the ability to divide into stem/progenitor cells, whereas this effect is antagonized by βAR antagonists propranolol, timolol, and ICI 118, 551 [33]. Consistently, ICI 118, 551 could reduce the myeloid cell numbers in the blood and spleen and lower the proliferation of splenic granulocyte macrophage progenitors (GMPs), indicating that β2AR signaling mediates the proliferation and myelopoiesis of GMPs [34]. In parallel, our data revealed that β-blockers eye drops delayed corneal wound healing through β2AR antagonism of limbal stem cells.

Ki67, localized in the nucleus, has been found negative in the G0 phase, whereas it increased during the G1 phase, and slight variation resulted in variable Ki67 levels in the G1 phase after cell cycle re-entry [35–37]. Therefore, Ki67 has been widely used as a proliferation marker [38]. In the present study, we found the expression of Ki67 was reduced in the corneas treated with levobunolol and ICI 118, 551, indicating that inhibition of β2AR led to a decrease in corneal epithelial cells in the G1 phase of the cell cycle. Moreover, β2AR exerts an effect on proliferation, mainly by activating signaling such as phosphorylation ERK1/2, cyclic adenosine monophosphate response element-binding protein (CREB), signal transducer, and activator of transcription 3 (STAT3), as well as the EGFR pathway which has been found to be activated during corneal epithelial wound healing in vivo [30, 39]. Our data revealed that CK3, CK14 and CK19, the phosphorylation of ERK1/2 and EGFR, was reduced by levobunolol and ICI 118, 551 treatment, suggesting that β2AR participated in the corneal wound healing process.

In clinical trials, both the β-blocker eye drops levobunolol (0.5%) and timolol (0.5%) applied twice daily for 4 weeks significantly decreased tear volume and corneal epithelial barrier function [18, 40]. In line with our results, previous studies showed that β-blocker eye drops interfere with corneal epithelial wound healing [19, 22]. Interestingly, we found that β-blocker eye drops were more influential in the corneal limbal stem cells than in the central epithelium. β2AR is predominantly found in membrane homogenates of the human iris-ciliary body and β1AR comprises approximately 10% of βAR in the entire iris-ciliary body [41]. Nonselective β1- and β2- antagonists, and relatively selective β1-antagonists have been used widely to treat ocular hypertension and glaucoma [42, 43]. In the cornea, β2AR is the predominant βAR, and β1AR comprises approximately 17% of βAR in the cornea [26]. Moreover, our results showed that the β1AR antagonist had no significant effect on the inhibition of corneal wound healing. Considering the fragile ocular surface of patients with glaucoma and the side effects of β-blocker eye drops on the ocular surface, non-selective β-blocker eye drops may not be the first choice for glaucoma treatment, whereas our results suggested that a selective β1AR antagonist, such as betaxolol or any other type of anti-glaucoma agent should be considered.

## Conclusions

β-blocker eye drops impaired corneal wound healing through inhibition the β2AR of limbal stem cells and resulted in the decrease of corneal epithelial regeneration-related signaling. Therefore, a selective β1AR antagonist might be a good choice for glaucoma treatment to avoid the ocular surface damage.

## Data Availability

The datasets used and/or analysed during the current study are available from the corresponding author on reasonable request.

## Abbreviations

β1AR: β1-adrenoceptor
β2AR: β2-adrenoceptor
IOP: Intraocular pressure
Ars: Adrenoceptors
GMPs: Granulocyte macrophage progenitors
CREB: Cyclic adenosine monophosphate response element-binding protein
STAT3: Activator of transcription 3

## References

[1] Graue-Hernández EO, Navas A, Ramírez-Miranda A, Holland EJ, Mannis MJ, Lee WB (2013). Toxic keratoconjunctivitis. Ocular surface disease: cornea, conjunctiva and tear film.

[2] Holdiness MR (2001). Contact dermatitis to topical drugs for glaucoma. Am J Contact Dermat.

[3] Xiang Y, Kobilka BK (2003). Myocyte adrenoceptor signaling pathways. Science..

[4] Crider JY, Sharif NA (2002). Adenylyl cyclase activity mediated by beta adrenoceptors in immortalized human trabecular meshwork and nonpigmented ciliary epithelial cells. J Ocul Pharmacol Ther.

[5] Wax MB, Molinoff PB (1987). Distribution and properties of beta-adrenergic receptors in human iris-ciliary body. Invest Ophthalmol Vis Sci.

[6] Ferrari-Dileo G (1988). Beta 1 and beta 2 adrenergic binding sites in bovine retina and retinal blood vessels. Invest Ophthalmol Vis Sci.

[7] Westfall TC (2009). Sympathomimetic drugs and adrenergic receptor antagonists. Encyclopedia Neuroence.

[8] Brooks AMV, Gillies WE (1992). Ocular β-blockers in Glaucoma management. Drugs Aging.

[9] Cinotti A, Cinotti D, Grant W, Jacobs I, Galin M, Silverstone D (1985). Levobunololvs timolol for open-angle glaucoma and ocular hypertension. Am J Ophthalmol.

[10] Halper LK, Johnson-Pratt L, Dobbins T, Hartenbaum D (2002). A comparison of the efficacy and tolerability of 0.5% timolol maleate ophthalmic gel-forming solution QD and 0.5% levobunolol hydrochloride BID in patients with ocular hypertension or open-angle glaucoma. J Ocul Pharmacol Ther.

[11] Shiuey Y, Eisenberg MJ (1996). Cardiovascular effects of commonly used ophthalmic medications. ClinCardiol..

[12] Sherwood MB, Grierson I, Millar L, Hitchings RA (1989). Long-term morphologic effects of antiglaucoma drugs on the conjunctiva and Tenon's capsule in glaucomatous patients. Ophthalmology..

[13] Herreras JM, Pastor JC, Calonge M, Asensio VM (1992). Ocular surface alteration after long-term treatment with an antiglaucomatous drug. Ophthalmology..

[14] Bonomi L, Zavarise G, Noya E, Michieletto S (1980). Effects of timolol maleate on tear flow in human eyes. Albrecht Von Graefes Arch KlinExpOphthalmol.

[15] Strempel I (1984). The influence of topical beta-blockers on the breakup time. Ophthalmologica..

[16] Kuppens EV, Stolwijk TR, de Keizer RJ, van Best JA (1992). Basal tear turnover and topical timolol in glaucoma patients and healthy controls by fluorophotometry. Invest Ophthalmol Vis Sci.

[17] McMahon CD, Shaffer RN, Hoskins HD, Hetherington J (1979). Adverse effects experienced by patients taking timolol. Am J Ophthalmol.

[18] Niiya A, Yokoi N, Matsumoto Y, Komuro A, Ishibashi T, Tomii S, He J (2000). Effect of beta-blocker eyedrops on corneal epithelial barrier function. Ophthalmologica..

[19] Haruta Y, Ohashi Y, Matsuda S (1997). Corneal epithelial deficiency induced by the use of beta-blocker eye drops. Eur J Ophthalmol.

[20] Liu GS, Trope GE, Basu PK (1990). Beta adrenoceptors and regenerating corneal epithelium. J Ocular pharmacol.

[21] Nam M (2021). Sun Woong Kim. Changes in corneal epithelial thickness induced by topical Antiglaucoma medications. J Clin Med.

[22] Dua HS, Azuara-Blanco A (2000). Limbal stem cells of the corneal epithelium. Surv Ophthal mol.

[23] Mastropasqua R, Agnifili L, Fasanella V, Curcio C, Brescia L, Lanzini M, Fresina M, Mastropasqua L, Marchini G (2015). Corneoscleral limbus in glaucoma patients: in vivo confocal microscopy and immunocytologicalstudy. Invest Ophthalmol Vis Sci.

[24] Heel RC, Brogden RN, Speight TM, Avery GS (1979). Atenolol: a review of its pharmacological properties and therapeutic efficacy in angina pectoris and hypertension. Drugs..

[25] Wenzel D, Knies R, Matthey M, Klein AM, Welschoff J, Stolle V (2009). beta(2)-adrenoceptor antagonist ICI 118,551 decreases pulmonary vascular tone in mice via a G(i/o) protein/nitric oxide-coupled pathway. Hypertension..

[26] Kawakita T, Shimmura S, Hornia A, Higa K, Tseng G, SC. (2008). Stratified epithelial sheets engineered from a single adult murine corneal/limbal progenitor cell. J Cell Mol Med.

[27] Qu Y, Lin H, Geng Z, Hui H, Liu Z, Wei L (2014). The phenotype study of murine cornealepithelial progenitor cell line TKE2. Chin J Cell Stem Cell (Electronic Edition).

[28] Yang L, Di G, Qi X, Qu M, Wang Y, Duan HY (2014). Substance P promotes diabetic corneal epithelial wound healing through molecular mechanisms mediated via the neurokinin-1 receptor. Diabetes..

[29] Xue Y, He J, Xiao C, Guo Y, Fu T, Liu J (2018). The mouse autonomic nervous system modulates inflammation and epithelial renewal after corneal abrasion through the activation of distinct local macrophages. Mucosal Immunol.

[30] Hu P, He J, Liu S, Wang M, Pan B, Wen Z (2016). ß2-adrenergic receptor activation promotes the proliferation of A549 lung cancer cells via the ERK1/2/CREB pathway. Oncol Rep.

[31] He JJ, Zhang WH, Liu SL, Chen YF, Liao CX, Shen QQ, Hu P (2017). Activation of beta-adrenergic receptor promotes cellular proliferation in human glioblastoma. Oncollett..

[32] Zeng H, Li H, Yue M, Fan Y, Cheng J, Wu X (2020). Isoprenaline protects intestinal stem cells from chemotherapy-induced damage. Br J Pharmacol.

[33] Masuda T, Nakagawa S, Boku S, Nishikawa H, Takamura N, Kato A (2012). Noradrenaline increases neural precursor cells derived from adult rat dentate gyrus through beta2 receptor. Prog Neuropsych Opharmacol Biol Psychiatry.

[34] Vasamsetti SB, Florentin J, Coppin E, Stiekema A, LC, Zheng KH, Muhammad UN. (2018). Sympathetic neuronal activation triggers myeloid progenitor proliferation and differentiation. Immunity..

[35] Gerdes J, Lemke H, Baisch H, Wacker HH, Schwab U, Stein H (1984). Cell cycle analysis of a cell proliferation-associated human nuclear antigen defined by the monoclonal antibody Ki-67. J Immunol.

[36] Sobecki M, Mrouj K, Camasses A, Parisis N, Nicolas E, Llères D (2016). The cell proliferation antigen Ki-67 organises heterochromatin. Elife..

[37] Sobecki M, Mrouj K, Colinge J, Gerbe F, Jay P, Krasinska L (2017). Cell-cycle regulation accounts for variability in Ki-67 expression levels. Cancer Res.

[38] Sun X, Kaufman PD (2018). Ki-67: more than a proliferation marker. Chromosoma..

[39] Zieske JD, Takahashi H, Hutcheon AE, Dalbone AC (2000). Activation of epidermal growth factor receptor during corneal epithelial migration. Invest Ophthalmol Vis Sci.

[40] Ishibashi T, Yokoi N, Kinoshita S (2003). Comparison of the effects of topical levobunololand timolol solution on the human ocular surface. Cornea..

[41] Wax MB, MolinoffPB. (1987). Distribution and properties of beta-adrenergic receptors in human irisciliary body. Invest Ophthalmol Vis Sci.

[42] Zimmerman TJ (1993). Topical ophthalmic beta blockers: a comparative review. J Ocul Pharmacol.

[43] Brooks AM, Gillies WE (1992). Ocular beta-blockers in glaucoma management. Clinical pharmacological aspects. Drugs Aging.

